# Supplementary material for: Anillin mediates unilateral furrowing during cytokinesis by limiting RhoA binding to its effectors
Source: J Cell Biol. 2025 Apr 22;224(6):e202405182. doi: 10.1083/jcb.202405182 (PMC12013513; doi:10.1083/jcb.202405182)
Supplement: Table S1 — contains C. elegans strains used in the study. [file jcb_202405182_tables1.docx]

**Table S1 contains *C. elegans* strains used in the study.**

| **Genotype** | **Reference** | **Strain name** |
| --- | --- | --- |
| Wild type | - | N2 |
| *nmy-2(cp52[nmy-2::mkate2 + LoxP unc-119(+) LoxP]) I; unc-119(ed3) III* | (Dickinson et al., 2017) | LP229 |
| *cyk-1::gfp* | (Reymann et al., 2016) | SWG004 |
| *oxTi179 II; unc-119(ed3) III* | (Frøkjær-Jensen et al., 2008) | EG8079 |
| *oxTi444 III; unc-119(ed3) III* | (Frøkjær-Jensen et al., 2008) | EG8080 |
| *oxTi177 IV; unc-119(ed3) III* | (Frøkjær-Jensen et al., 2008) | EG8081 |
| *let-502(mc74[GFP::let-502]) I; (zuIs151 [nmy-2::NMY-2-mRFP; unc-119(+)])* | (Bell et al., 2020) | EM328 |
| *nmy-2(cp13[nmy-2::gfp + LoxP]) I; OD56 (mCherry::histone H2B; ltIs44[pie-1p::mCherry::PH(PLC1delta1)+unc-119(+)]* | (Lebedev et al., 2023) | ZAN23 |
| *it-rho-1(syb1605)/nT1 IV* | This study | ZAN350 |
| *nmy-2(cp52[nmy-2::mkate2 + LoxP unc-119(+) LoxP]) I; unc-119(ed3) III; ani-1(mon7[mNeonGreen^3xFlag::ani-1]) III* | (Lebedev et al., 2023) | ZAN351 |
| *nmy-2(cp52[nmy-2::mkate2 + LoxP unc-119(+) LoxP]) I; unc-119(ed3) III?; it-rho-1(syb1605)/nT1 IV* | This study | ZAN358 |
| *Si192[pEZ379; pani-1::GFP::ANI-1^WT^; cb-unc-119(+)]II; unc-119(ed3) III?* | (Lebedev et al., 2023) | ZAN373 |
| *Si194[pEZ389; pani-1::GFP::ANI-1^Link+C-term^ (∆48-460 AA); cb-unc-119(+)]II; unc-119(ed3) III?* | (Lebedev et al., 2023) | ZAN376 |
| *Si200[pEZ391; pani-1::GFP::ANI-1^C-term^ (∆48-680 AA); cb-unc-119(+)] II; unc-119(ed3) III?* | (Lebedev et al., 2023) | ZAN382 |
| *Si203[pEZ419; pani-1::GFP::ANI-1^N-term+Link^ (1-763 AA); cb-unc-119(+)] II; unc-119(ed3) III?* | (Lebedev et al., 2023) | ZAN386 |
| *Si204[pEZ422; pani-1::GFP::ANI-1^Linker-CX^ (1-47AA, 441-763 AA-PBS-CX); cb-unc-119(+)] II; unc-119(ed3) III* | (Lebedev et al., 2023) | ZAN387 |
| *nmy-2(cp52[nmy-2::mkate2 + LoxP unc-119(+) LoxP]) I; Si200[pEZ391; pani-1::GFP::ANI-1^C-term^ (∆48-680 AA); cb-unc-119(+)] II; unc-119(ed3) III?* | This study | ZAN388 |
| *nmy-2(cp52[nmy-2::mkate2 + LoxP unc-119(+) LoxP]) I; Si192[pEZ379; pani-1::GFP::ANI-1^WT^; cb-unc-119(+)] II; unc-119(ed3) III?* | This study | ZAN389 |
| *nmy-2(cp52[nmy-2::mkate2 + LoxP unc-119(+) LoxP]) I; Si194[pEZ389; pani-1::GFP::ANI-1^Link+C-term^ (∆48-460 AA); cb-unc-119(+)] II; unc-119(ed3) III?* | This study | ZAN396 |
| *nmy-2(cp52[nmy-2::mkate2 + LoxP unc-119(+) LoxP]) I; Si203[pEZ419; pani-1::GFP::ANI-1^N-term+Link^ (1-763 AA); cb-unc-119(+)] II; unc-119(ed3) III?* | This study | ZAN404 |
| *Si212[pEZ443; pani-1::GFP::ANI-1^C-term-RBM^ (∆48-680 AA), A789D, E807K; cb-unc-119(+)] II; unc-119(ed3) III?* | This study | ZAN405 |
| *nmy-2(cp52[nmy-2::mkate2 + LoxP unc-119(+) LoxP]) I; Si212[pEZ443; pani-1::GFP::ANI-1^C-term-RBM^ (∆48-680 AA), A789D, E807K; cb-unc-119(+)] II; unc-119(ed3) III?* | This study | ZAN412 |
| *Si217[pEZ448; pani-1::3xFLAG::ANI-1^C-term^ (∆48-680 AA); cb-unc-119(+)] III; unc-119(ed3) III?* | This study | ZAN428 |
| *nmy-2(cp52[nmy-2::mkate2 + LoxP unc-119(+) LoxP]) I; Si204[pEZ422; pani-1::GFP::ANI-1^Linker-CX^ (1-47AA, 441-763 AA-PBS-CX); cb-unc-119(+)] II; unc-119(ed3) III?* | This study | ZAN429 |
| *Si219[pEZ450; pani-1::3xFLAG::ANI-1^WT^; cb-unc-119(+)] II; unc-119(ed3) III?* | This study | ZAN431 |
| *nmy-2(cp52[nmy-2::mkate2 + LoxP unc-119(+) LoxP]) I; unc-119(ed3) III?; Si220[pEZ456; pani-1::3xFLAG::ANI-1^Link+C-term^ (∆48-460 AA); cb-unc-119(+)] IV* | This study | ZAN432 |
| *nmy-2(cp52[nmy-2::mkate2 + LoxP unc-119(+) LoxP]) I; Si219[pEZ450; pani-1::3xFLAG::ANI-1^WT^; cb-unc-119(+)] II; unc-119(ed3) III?* | This study | ZAN433 |
| *unc-119(ed3) III; Si220[pEZ456; pani-1::3xFLAG::ANI-1^Link+C-term^ (∆48-460 AA); cb-unc-119(+)] IV* | This study | ZAN434 |
| *nmy-2(cp52[nmy-2::mkate2 + LoxP unc-119(+) LoxP]) I; Si217[pEZ448; pani-1::3xFLAG::ANI-1^C-term^ (∆48-680 AA); cb-unc-119(+)] III; unc-119(ed3) III?* | This study | ZAN436 |
| *nmy-2(cp52[nmy-2::mkate2 + LoxP unc-119(+) LoxP]) I; Si203[pEZ419; pani-1::GFP::ANI-1^N-term+Link^ (1-763 AA); cb-unc-119(+)] II; Si217[pEZ448; pani-1::3xFLAG::ANI-1^C-term^ (∆48-680 AA); cb-unc-119(+)] III; unc-119(ed3) III?* | This study | ZAN437 |
| *Si222[pEZ455; pani-1::3xFLAG::ANI-1^N-term+Link^ (1-763 AA); cb-unc-119(+)] II; unc-119(ed3) III?* | This study | ZAN439 |
| *nmy-2(cp52[nmy-2::mkate2 + LoxP unc-119(+) LoxP]) I; Si222[pEZ455; pani-1::3xFLAG::ANI-1^N-term+Link^ (1-763 AA); cb-unc-119(+)] II; unc-119(ed3) III?* | This study | ZAN440 |
