# Supplementary material for: Anillin mediates unilateral furrowing during cytokinesis by limiting RhoA binding to its effectors
Source: J Cell Biol. 2025 Apr 22;224(6):e202405182. doi: 10.1083/jcb.202405182 (PMC12013513; doi:10.1083/jcb.202405182)
Supplement: Table S2 — shows the information about the dsRNA used in the study. [file jcb_202405182_tables2.docx]

**Table S2** shows the information about the dsRNA used in the study. The T7 sequence is underlined.

| Gene | Oligonucleotide 1 | Oligonucleotide 2 | Source | RNAi condition | Concentration [µg/µl] |
| --- | --- | --- | --- | --- | --- |
| *cyk-1* | TAATACGACTCACTATAGGTTGGAGTTCGATGCAGAAGA | TAATACGACTCACTATAGGTTGCCTTGTCAGGAACTGAA | cDNA | injection | 0.5-1.0 |
| *ani-1* | TAATACGACTCACTATAGGAGCCGGAGTTGGAAAGCTG | TAATACGACTCACTATAGGCCTATTCTTTTCCAAACGTTGC | genomic DNA | injection | 0.35-0.7 |
| *rho-1* | TAATACGACTCACTATAGGGTCGAACTTGCTCTATGGGAT | TAATACGACTCACTATAGGGCAGAGCACTCCAAATATGC | genomic DNA | injection | 1.0 |
| *ect-2* | TAATACGACTCACTATAGGATTCTCGAACTTCACGAAAGG | TAATACGACTCACTATAGGGACGAGTAGAAGAATCTCCC | cDNA | injection | 0.68 |
| *rga-3/4* | TAATACGACTCACTATAGGGCAACGCGTCGAAACATCG | TAATACGACTCACTATAGGGTTGGAGTGGCAGTTGGAGTG | cDNA | injection | 0.5 |
| *perm-1* | AATGTTTATGAACCCGAGCG | TTCACGCAGTTGTTGACACA | (Carvalho et al., 2011) | feeding | N.A. |
| *ani-1* | CATGTTCACTGACAACTGGGATA | CAAACTCAATGGAGAGGACAATC | Bioscience | feeding | N.A. |
