# Supplementary material for: Anillin mediates unilateral furrowing during cytokinesis by limiting RhoA binding to its effectors
Source: J Cell Biol. 2025 Apr 22;224(6):e202405182. doi: 10.1083/jcb.202405182 (PMC12013513; doi:10.1083/jcb.202405182)
Supplement: Table S3 — contains recombinant DNA used in this study. [file jcb_202405182_tables3.docx]

**Table S3 contains recombinant DNA used in this study.**

| **Plasmid name** | **Reference** | **Plasmid number** |
| --- | --- | --- |
| pGEX-4T-1: GST-RHO-1^WT^ (1-188 AA) | This study | pEZ162 |
| pET19b: 10xHis-RHO-1^Q63L^ (1-188 AA) | This study | pEZ222 |
| pET19b: 10xHis-RHO-1^WT^ (1-188 AA) | This study | pEZ235 |
| pGEX-4T-1 | - | pGEX-4T-1 |
| pGEX-4T-1: GST-ANI-1^Linker^ (460-775 AA) | This study | pEZ397 |
| pGEX-4T-1: GST-ANI-1^C-term^ (681-1159 AA) | This study | pEZ437 |
| pGEX-4T-1: GST-ANI-1^C-term-RBM^ (681-1159 AA, A789D, E807K) | This study | pEZ444 |
| pGEX-4T-1: GST-ANI-1^Link+C-term^ (460-1159 AA) | This study | pEZ457 |
| pCFJ350: pani-1::gfp::ani-1^C-term-RBM^ (∆48-680 AA, A789D, E807K)::ani-1 | This study | pEZ443 |
| pCFJ350: pani-1::3xFLAG::ani-1^C-term^ (∆48-680 AA)::ani-1 | This study | pEZ448 |
| pCFJ350: pani-1::3xFLAG::ani-1^WT^ (1-1159 AA)::ani-1 | This study | pEZ450 |
| pCFJ350: pani-1::3xFLAG::ani-1^N-term+Link^ (1-763 AA)::ani-1 | This study | pEZ455 |
| pCFJ350: pani-1::3xFLAG::ani-1^Link+C-term^ (∆48-460 AA)::ani-1 | This study | pEZ456 |
