# Supplementary material for: Anillin mediates unilateral furrowing during cytokinesis by limiting RhoA binding to its effectors
Source: J Cell Biol. 2025 Apr 22;224(6):e202405182. doi: 10.1083/jcb.202405182 (PMC12013513; doi:10.1083/jcb.202405182)
Supplement: Table S4 — contains DNA oligos used for cloning in this study. [file jcb_202405182_tables4.docx]

**Table S4 DNA oligos used for cloning in this study**

| **ID** | **Sequence 5’-3’** | **Related to plasmid #** |
| --- | --- | --- |
| EZ563 | ATCGTCAGTCACTTGCTCTTCTTTTTCTTCTTC | pEZ162 |
| EZ564 | AGAAGAGCAAGTGACTGACGATCTGCCTCGCG | pEZ162 |
| EZ573 | CGGAATTCCCGATGGCTGCGATTAGAAAGAAG | pEZ162 |
| EZ574 | ATCGCAGCCATCGGGAATTCCGGGGATCCAC | pEZ162 |
| EZ309 | CTCTATGGGATACAGCTGGACTCGAGGACTATGATCGTCTG | pEZ222 |
| EZ310 | CAGACGATCATAGTCCTCGAGTCCAGCTGTATCCCATAGAG | pEZ222 |
| EZ816 | GCAAGTGACTGCCGGCTGCTAACAAAGCCC | pEZ222, pEZ235 |
| EZ817 | ATCGCAGCCATATCCTCGAGCATATGCTTGTC | pEZ222, pEZ235 |
| EZ818 | TGCTCGAGGATATGGCTGCGATTAGAAAGAAG | pEZ222, pEZ235 |
| EZ821 | TTAGCAGCCGGCAGTCACTTGCTCTTCTTTTTC | pEZ222, pEZ235 |
| EZ1467 | AAGCACCTGTTCCCCGTAGTG | pEZ397 |
| EZ1489 | GCGAGGCAGATCGTCAGTCACGCTCGCTTGATACGATCG | pEZ397 |
| EZ1490 | TGACTGACGATCTGCCTCG | pEZ397 |
| EZ1495 | ACTACGGGGAACAGGTGCTTCGGGAATTCCGGGGATCCAC | pEZ397 |
| EZ1487 | GATGACGATGCAACAACACAATTTC | pEZ437, pEZ444 |
| EZ1580 | TGTTGTTGCATCGTCATCCGGGAATTCCGGGGATCCA | pEZ437, pEZ444 |
| EZ1581 | GAGGCAGATCGTCAGTCACTGATTAACAATCGGATTTCTCC | pEZ437 |
| EZ1582 | TGACTGACGATCTGCCTCGC | pEZ437 |
| EZ1605 | CATTGCCCATTGTGCTTTAAATTCCTCACGCG | pEZ444, pEZ443 |
| EZ1606 | CGCGTGAGGAATTTAAAGCACAATGGGCAATG | pEZ444, pEZ443 |
| EZ793 | GCTGTTGAGATCCAGTTCGATG | pEZ457, pEZ443, pEZ448, pEZ450, pEZ455, pEZ456 |
| EZ794 | CATCGAACTGGATCTCAACAGC | pEZ457, pEZ443, pEZ448, pEZ450, pEZ455, pEZ456 |
| EZ1457 | AAAGCGGCCGCTCTCTTCTTCTTCCGGCTCATAG | pEZ457 |
| EZ1646 | GAAATTGTGTTGTTGCATCGTCATC | pEZ457 |
| EZ1094 | GACGGGAACTACAAGACACG | pEZ443 |
| EZ1095 | CGTGTCTTGTAGTTCCCGTC | pEZ443 |
| EZ1603 | CCCGGGCCAATATCATATCTCTCTTGGATTG | pEZ443 |
| EZ1604 | CAATCCAAGAGAGATATGATATTGGCCCGGG | pEZ443 |
| EZ1623 | CTTGTCGTCGTCGTCCTTGTAGTCACGCTTATCGTCATCGTCTTTATAATCCATTGTATTTACAGTGTAGTTCTG | pEZ448 |
| EZ1624 | TACAAGGACGACGACGACAAGCGTGATTACAAGGATGACGATGACAAGAGAGGGGATCAATTCGATTCTCTAATG | pEZ448 |
| EZ1419 | ACCTCCGCCGGCGGTCTATTTTTTTCCAAGCGCTG | pEZ450 |
| EZ1420 | AAAATAGACCGCCGGCGGAGGTTATTGAAG | pEZ450 |
| EZ1466 | TGTTGGAGATGCAACTGAAAATC | pEZ450, pEZ456 |
| EZ1645 | TTGATTTTCAGTTGCATCTCCAAC | pEZ450 |
| EZ1540 | CGAATTGTCGAGTAGTCCCCCCCTTTTTTGC | pEZ455 |
| EZ1541 | GGGGACTACTCGACAATTCGTGCTCCATTC | pEZ455 |
| EZ1474 | ATTTTCAGTTGCATCTCCAACAAAGCACCTGTTCCCCGTAGTG | pEZ456 |
