# Supplementary figures and images for: Anillin mediates unilateral furrowing during cytokinesis by limiting RhoA binding to its effectors
Source: J Cell Biol. 2025 Apr 22;224(6):e202405182. doi: 10.1083/jcb.202405182 (PMC12013513; doi:10.1083/jcb.202405182)

B

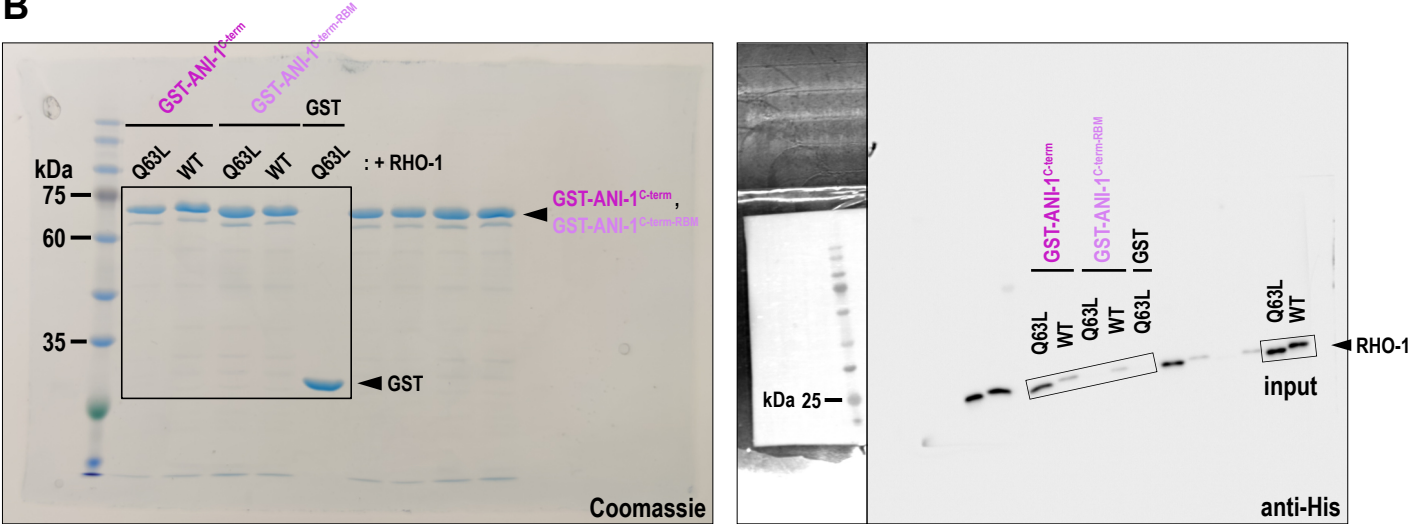

C

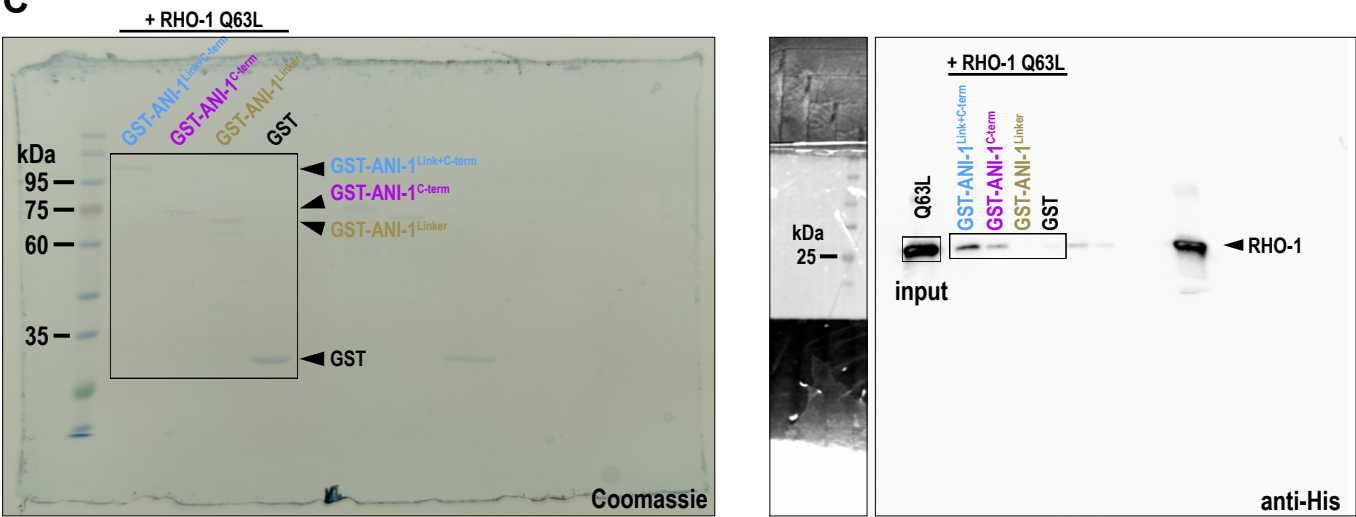

Supplement: SourceData F2 — is the source file for Fig. 2. [file jcb_202405182_sourcedataf2.pdf]

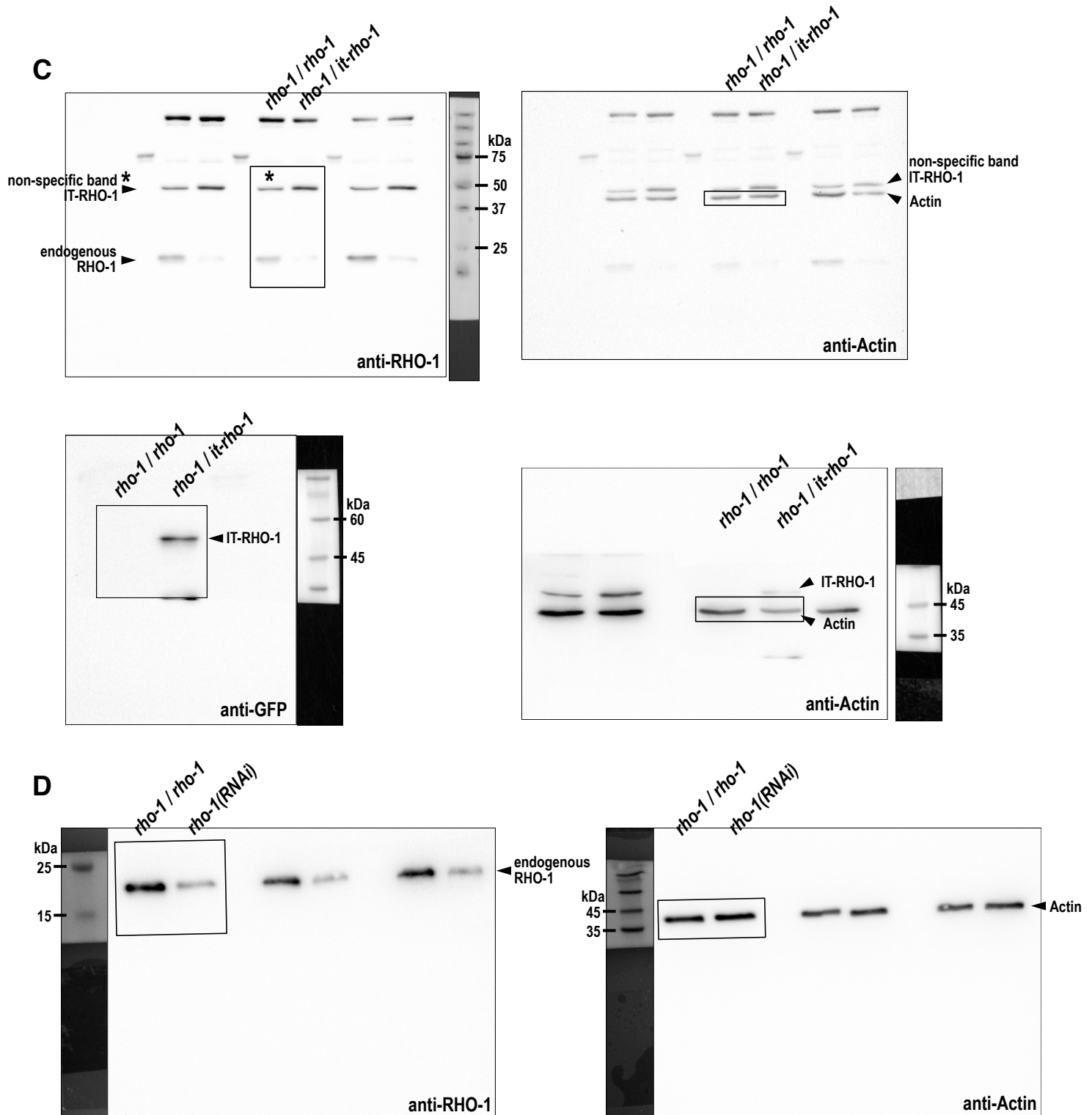

Supplement: SourceData FS2 — is the source file for Fig. S2. [file jcb_202405182_sourcedatafs2.pdf]

**E**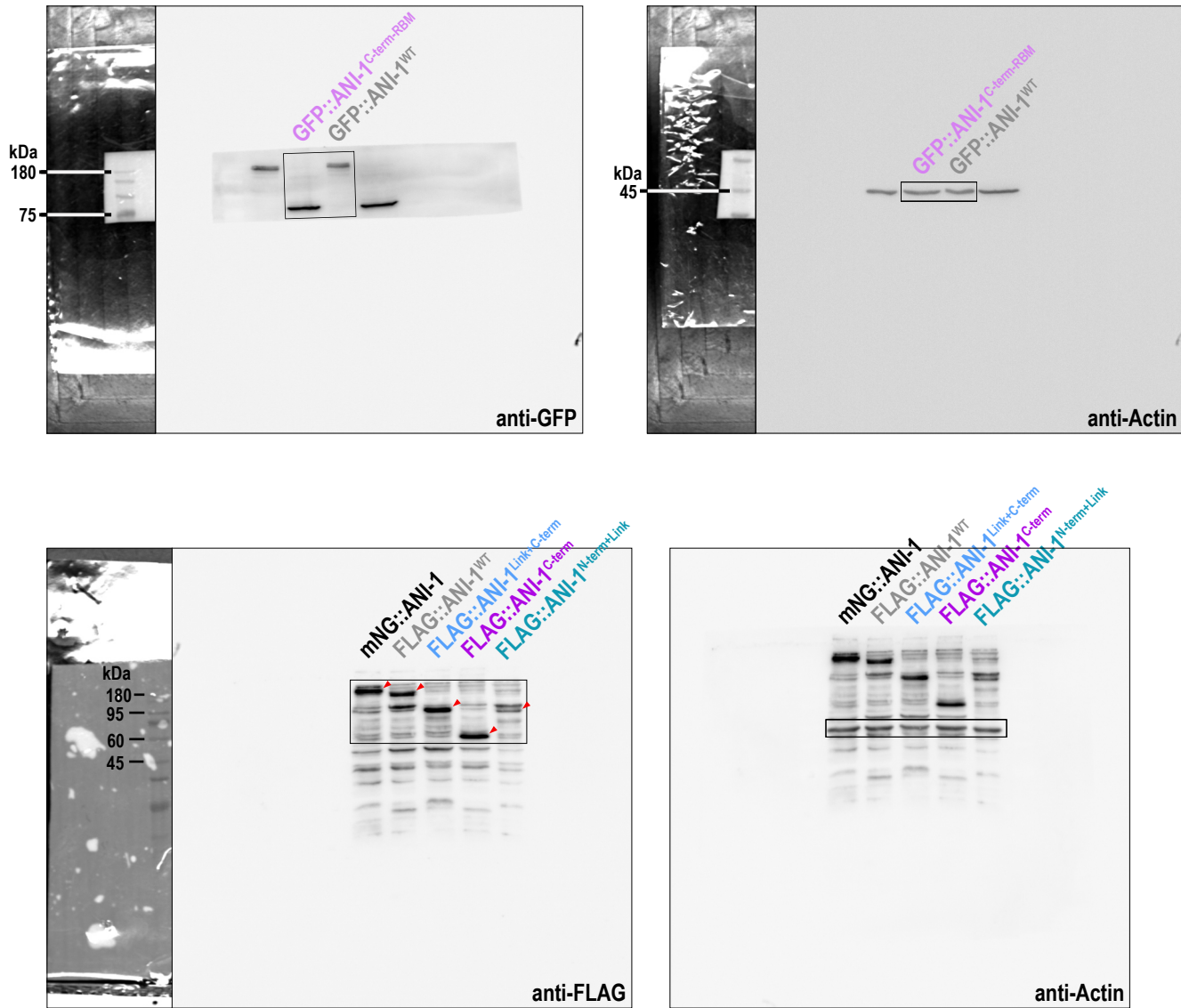

Supplement: SourceData FS4 — is the source file for Fig. S4. [file jcb_202405182_sourcedatafs4.pdf]
